# Evaluation of the clinical results of posterior cruciate ligament reconstruction -a comparison between the use of the bone tendon bone and semitendinosus and gracilis tendons-

**DOI:** 10.1186/1758-2555-4-30

**Published:** 2012-08-29

**Authors:** Yuichiro Maruyama, Katsuo Shitoto, Tomonori Baba, Kazuo Kaneko

**Affiliations:** 1 Department of Orthopaedic Surgery, Juntendo University Urayasu Hospital, 2-1-1 Tomioka, Urayasu, Chiba prefecture, Postal code 279-0021, Japan; 2 Department of Orthopaedic Surgery, Juntendo University School of Medicine, 2-1-1 Hongo Bunkyo-ku, Tokyo, Postal code 113-8421, Japan

## Introduction

The techniques for reconstruction of the posterior cruciate ligament (PCL) are still being developed. There are some options when choosing a graft for PCL reconstruction. The bone tendon bone (BTB) and semitendinosus and gracilis tendons (STG) are widely used, but both have advantages and disadvantages. Each result after ligament reconstruction has been reviewed. However, few studies, in which the same surgeon has conducted the same rehabilitation program using the same bone tunnel, have compared postoperative results between BTB and STG. It remains to be clarified whether the results differ according to graft materials and their fixation between the two techniques [1-5].

We performed anterolateral single bundle reconstruction of the PCL using BTB and STG in a total of 30 cases, and the courses of the patients were observed for over one year. The postoperative results of PCL reconstruction using BTB and STG were compared and studied retrospectively. The purpose of the present study was to clarify the features of the two surgical methods and to explore the problems associated with these procedures and measures that could be used to improve them.

## Materials and methods

Thirty patients who had undergone PCL reconstruction and had been followed-up for more than twelve months were the subjects of this study. Our retrospective study included 14 patients treated with the transtibial technique using bone patellar tendon bone (BTB group) and 16 patients who underwent the transtibial anterolateral single-bundle technique using semitendinosus and gracilis tendons (STG group).

The study population comprised 13 men and 1 woman in the BTB Group, and 12 men and 4 women in the STG Group. The mean length of follow-up was 35.0 ± 40.0 months (12–156) in the BTB group and 23.5 ± 14.7 months (12–62) in the STG group. The mean time from injury to surgery was 24.1 ± 33.3 months (2–125) in the BTB group and 23.4 ± 37.5 months (1–144) in the STG group. At the time of the operation, the mean age was 31.1 ± 7.7 years (19–43) in the BTB group and 35.1 ± 9.4 years (19–54) in the STG group. The cause of injury included 4 motor vehicle accidents, 6 sports injuries, and 4 accidental fall in the BTB group and 7 motor vehicle accidents, 3 sports injuries, and 6 accidental falls in the STG group. Other associated ligamentous injuries; i.e., posterolateral corner laxities (6 cases), were treated with grafts from the iliotibial tract (2 cases) or hamstrings tendons (4 cases) (Table1).

**Table 1:** Profile of patients

	**BTB group**	**STG group**
Average time from injury to surgery	24.1 ± 33.3 months (2–125)	23.4 ± 37.5 months (1–144)
Average age at surgery	31.1 ± 7.7 years(19–43)	35.1 ± 9.4 years(19–54)
Average length of follow-up	35.0 ± 40.0 months (12–156)	23.5 ± 14.7 months (12–62)
Cause of injury		
Motor-vehicle accident	4	7
Sports	6	3
Accidental fall	4	6
Other ligamentous injury		
(posterolateral corner laxities)	4	2

### Clinical evaluation

**Figure 1:**
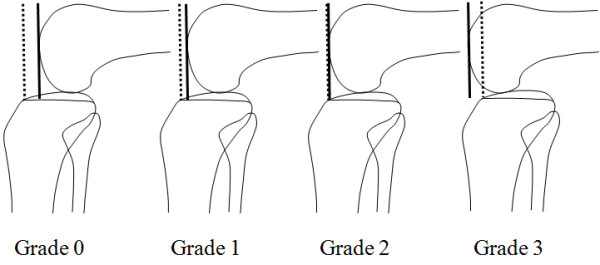
Posterior drawer test: This is done by determining the distance of the medial tibial plateau from the medial femoral condyle at 90˚ flexion while a posterior load is applied to the tibia.

The posterior drawer test was performed with the knee at 90° of flexion and the tibia in a neutral position. This is done by determining the distance of the medial tibial plateau from the medial femoral condyle. The tibia is normally located approximately 1 cm anterior to the femoral condyles in a resting position (Grade 0). Patients with Grade 1 injuries have a palpable but diminished step off. Patients with Grade 2 injuries have lost their step off, but their medial tibia plateau cannot be pushed beyond the medial femoral condyle. Patients with Grade 3 injuries have lost their medial step off, but their medial tibial plateau can be pushed beyond the medial femoral condyle (Figure1).

**Figure 2:**
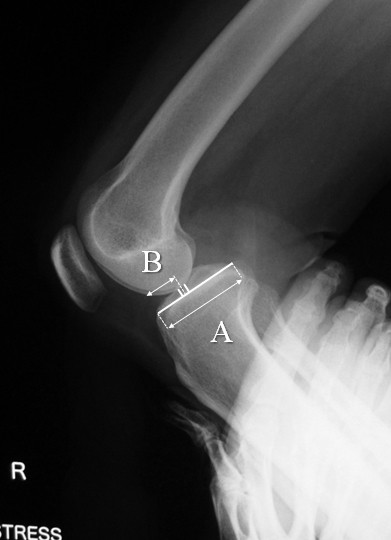
Mid-point displacement rate: When the anteroposterior diameter of the tibial plateau was taken as A and the anterior distance of the midpoint of a perpendicular line drawn to A from the lowest point of the medial and lateral femoral condyles was taken as B, and B/A x 100 was 45% or less, PCL injury was diagnosed.

Stress radiographs were analyzed according to the midpoint displacement rate, as described by Murase et al. [6]. In this method, the lateral view was imaged with the knee at 90° of flexion and under the maximum backward manual load. When the anteroposterior diameter of the tibial plateau was taken as A and the anterior distance of the midpoint of a perpendicular line drawn to A from the lowest point of the medial and lateral femoral condyles was taken as B, and B/A was 45% or less, PCL injury was diagnosed (Figure2).

A subjective clinical assessment was performed using the Lysholm score. Patients were excluded if their follow-up lasted less than 1 year. All PCL reconstructions were performed by one surgeon (Y.M.). Statistical analysis was performed using the unpaired t - test (2-tails).

The KT-2000 arthrometer was used to evaluate the side-to-side difference of anteroposterior laxity at 30 degrees of flexion in the BTB group(8 cases) and the STG group(10 cases).

### Operative technique

BTB group: The patients underwent an arthroscopically assisted anterolateral single-bundle PCL reconstruction. A transtibial guide pin was placed slightly lateral of the tibial footprint from the anterolateral cortex of the proximal part of the tibia. Its position was verified with intraoperative fluoroscopy. Then, a tibial tunnel of 10 mm in diameter was drilled. A femoral guide pin was inserted from the medial epicondyle at the two o’clock position in the right knee, 8 to 9 mm proximal to the articular junction. A femoral tunnel of 10 mm in diameter was then created. A bone patellar tendon bone (BTB) autograft of 9 mm in diameter was passed through the tibial tunnel and into the femoral tunnel. Aperture fixation of a bone plug was performed as close as possible to the exit of the tibial bone tunnel. At the time of the graft fixation, Knee position is 10 degrees in flexion. The graft was tensioned with manual maximum stress and fixed with cannulated interference screws (RCI, Smith & Nephew) to the femoral and tibial sides with the outside-in technique.

STG group: Tibial and femoral tunnels were made in the same manner as described for the BTB group. Suture disc was attached to the quadrupled hamstring tendon autograft for femoral fixation. The prepared graft of 9 or 10 mm in diameter was pulled through the femoral tunnel and into the tibial tunnel. The graft was then tensioned with manual maximum stress and fixed with the post screw technique to the tibia. Knee position is 10 degrees in flexion same as BTB group.

### Postoperative rehabilitation protocol

The rehabilitation program and weight bearing period were the same for the 2 groups. A knee brace was applied postoperatively with the knee in full extension. A functional PCL brace was fitted at 2 to 12 postoperative weeks. Partial weight bearing using 2 crutches was allowed from the next operative day. Weight bearing was gradually increased to full weight bearing at 3 weeks. Full ADL was allowed from 3 months. Jogging and low impact sports began at 6 months. A full return to sports was allowed from 8 to 12 months depending on the patient.

### Ethical approval and consent

Written informed consent was obtained from the patient for publication of this report and any accompanying images.

## Results

**Figure 3:**
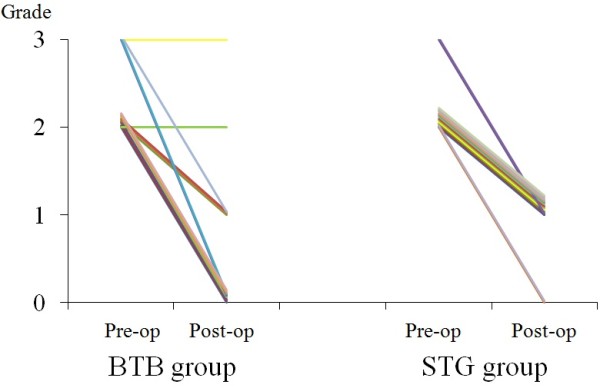
Clinical results of the posterior drawer test in pre- and postoperative BTB and STG groups.

In the BTB group, on the Posterior drawer test, 7 knees (50%) were Grade 0, and one knee each was Grade 2 and 3, respectively. In the STG group, only 2 knees were Grade 0, and all of the others were Grade 1.In the BTB group, favorable stability was achieved in half of cases, but unfavorable cases were also present. In the STG group, slight elongation was noted in all cases, but none of it was markedly unfavorable (Figure3).

**Figure 4:**
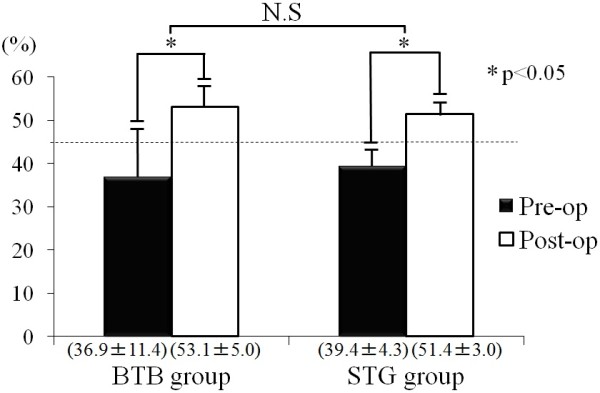
Clinical results of the mid-point displacement rate in pre- and postoperative BTB and STG groups.

On stress X-ray radiographic measurement, the value was improved from 36.9% before surgery to 53.1% after surgery in the BTB group and from 39.4 to 51.4% in the STG group, showing no significant difference between the groups (Figure4).

**Figure 5:**
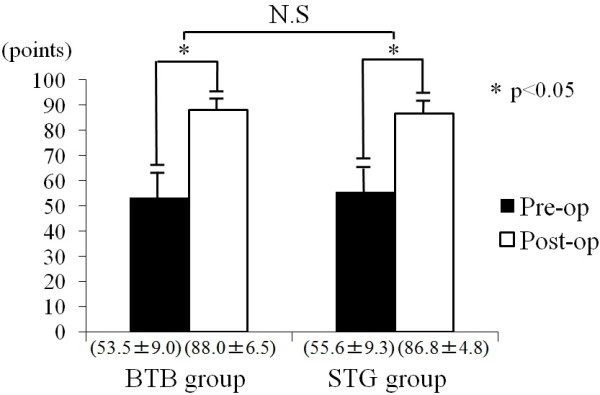
Functional results of the Lysholm score in pre- and postoperative BTB and STG groups.

Similarly, the Lysholm score was improved from 53.5 points before surgery to 88.0 points after surgery in the BTB group and from 55.6 to 86.8 points in the STG group, showing no significant difference between the groups (Figure5).

The mean side-to-side difference of the anteroposterior laxity as measured with KT-2000 arthrometer were improved from 5.5 mm before surgery to 1.6 mm after surgery in the BTB group and from 5.6 mm to 1.8 mm in the STG group. However, we excluded this data from the object of the final evaluation because it was not all cases.

Patients with combined PCL and posterolateral corner laxities (6 cases) who simultaneously underwent reconstruction with modified Larson method showed improvement from 33.5% before surgery to 52.4% after surgery on stress X-ray radiographic measurement, there was no difference from the patients with PCL reconstruction alone.

**Figure 6:**
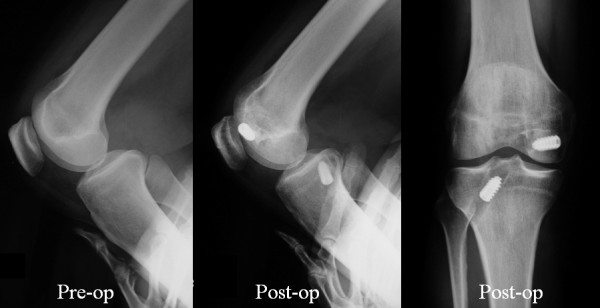
**Case 1 (BTB group).** A 48-year-old male was injured in a traffic accident. The preoperative posterior drawer test was Grade 2, mid-point displacement rate was38.5%, and Lysholm score was 65 points. At 28 months after surgery, his results for the above parameters were Grade 1, 50.2%, and 93 points, respectively.

Typical case 1 (BTB group): A 48-year-old male was injured in a traffic accident. His clinical characteristics were as follows: posterior drawer test, Grade 2; midpoint displacement rate, 38.5%; and Lysholm score, 65 points. At 28 months after surgery, his results for the above parameters were Grade 1, 50.2%, and 93 points, respectively (Figure6).

**Figure 7:**
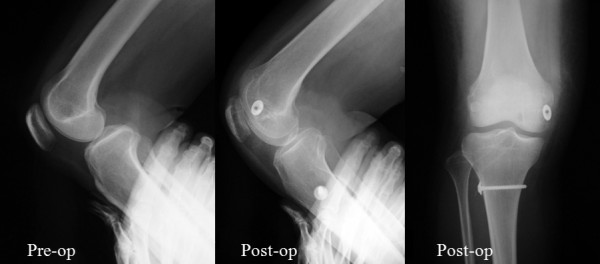
**Case 2 (STG group).** A 35-year-old female was injured due to a fall. The posterior drawer test was Grade 2, mid-point displacement rate was 32.5%, and Lysholm score was 60 points. At 17 months after surgery, her results for the above parameters were Grade 1, 51.0%, and 87 points, respectively.

Case 2 (STG group): A 35-year-old female was injured by a fall. Her clinical characteristics were as follows: posterior drawer test, Grade 2; midpoint displacement rate, 32.5%; and Lysholm score, 60 points. At 17 months after surgery, her results for the above parameters were Grade 1, 51.0%, and 87 points, respectively (Figure7).

## Discussion

Although the current outcomes of PCL reconstruction are unsatisfactory, it is often necessary to resolve problems associated with daily living activities and sports motions.

Our indications for surgery were cases categorized as Grade 2 or 3 on the posterior drawer test that were under conservative treatment and involved patients suffering from problems associated with daily living activities and functional and sport motions.

In transtibial single-bundle reconstruction using BTB or STG, problems including killer turns and postoperative time-course elongation remain. It remains to be clarified whether the results differ according to graft materials and their fixation between the two techniques.

All surgical procedures were performed during the same period, but the surgical procedure was not randomly selected. Rather, it was decided after consultation between the operator and patient.

Harner et al. classified graft choices for PCL reconstruction into autografts (BTB, STG, and quadriceps tendon) and allografts (Achilles tendon and BTB). The approaches were classified into single and double bundles, and graft placements were classified into the tibial tunnel and tibial inlay methods [7]. In Japan, autografts of the BTB and hamstring are generally used.

Regarding the approach, Kohen et al., systematically reviewed biomechanical and clinical studies of PCL reconstruction involving single and double bundles and concluded that at present it is unclear whether its outcomes are superior to those of previous methods [8].

Since Berg et al [9]. first reported it in 1995, the tibial-inlay method has been considered to be theoretically advantageous in many reports from Western countries such as that reported by Wind et al [10]. because it does not produce killer turns and more closely duplicates the normal PCL anatomy [11-13]. However, it has not yet become common in Japan because allografts cannot be freely used and posture changes during surgery are complex.

Therefore, we performed conventional anterolateral transtibial single-bundle reconstruction of the PCL using BTB and STG until now, so we compared the outcomes of both methods with the aim of minimizing complications.

When the BTB is used as the graft source, the fixation force is strong because the graft is fixed bone-to-bone with interference screw, which is advantageous; however, it can cause the following problems: muscle weakness of the graft-harvest region, pain around the patellofemoral joint, and wear of the graft caused by sharp angulation at the posterior opening of the tibial tunnel (killer turn). Regarding STG, hamstring tendon grafts can be harvested easily and the soft tissue end of the graft allows it to be easily passed through the tunnel, but graft fixation is inferior, the tendency for graft elongation over time has been reported. The same risk of killer turn is present also in the use of the STG graft, but STG involves eight bundles, and, hence, pressure is estimated to be dispersed even if the angle suddenly changes.

In the BTB group, the grade was unchanged in 2 cases: a Grade-3 case and a Grade-2 case, suggesting the presence of re-rupture due to wearing or elongation of the graft. However, the other outcomes of the BTB group were relatively favorable.

Therefore, we have attempted to minimize the problems of killer turn at the tibial tunnel by creating an anterolateral tibial bone tunnel, chamfering a tunnel exit as much as possible, preparing a cylindrical bone block into a column in order to fill the space between the graft and tunnel wall, and placing the bone fragment close to the posterior opening of the tibial tunnel [14,15].

In this study, the subjects included patients who simultaneously underwent treatment for posterolateral corner laxities, but there was no difference in the outcome between these patients and those who underwent PCL reconstruction alone.

Since the study was not prospective, short follow-up period, and the number of patients was small, precise comparison of the outcome is impossible at present, and so further evaluation is necessary.

## Conclusion

1. We compared the clinical results of two groups treated with anterolateral single-bundle reconstruction of the posterior cruciate ligament using the BTB or STG method with short term follow-up(a minimum of 12 months).

2. Post-operative outcome of the BTB group was relatively favorable except 2 cases. Excellent stability and fair results were obtained in the BTB group. The STG group showed slight residual knee laxity in all cases, but there were no complete failures.

3. Several techniques have been advocated to minimize the problem of killer turn in the BTB method.

## Competing interests

The authors declare that they have no competing interests.

## Authors’ contributions

YM drafted the manuscript. KS, TB and KK contributed to study design and manuscript sturucture. All authors read and approved the final manuscript.

## Author details

^1^Department of Orthopaedic Surgery, Juntendo University Urayasu Hospital, 2-1-1 Tomioka, Urayasu, Chiba prefecture Postal code 279-0021, Japan.^2^Department of Orthopaedic Surgery, Juntendo University School of Medicine, 2-1-1 Hongo Bunkyo-ku, Tokyo Postal code 113-8421, Japan.

## References

[B1] Hemans S Corten K Bellemans J Long-term results of isolated anterolateral bundle reconstructions of the posterior cruciate ligament, A6- to 12-year follow-up study Am J Sports Med 2009 37 1449 1507 10.1177/0363546509333479 19451096

[B2] Wiley WB Askew MJ Melby A Noe DA Kinematics of the Posterior cruciate ligament/posterior corner-injured knee after reconstruction by single- and double-bundle intra-articular grafts Am J Sports Med 2006 34 741 747 16382008 10.1177/0363546505282615

[B3] Chan YS Yang SC Chang CH Chen AC Yuan LJ Hsu KY Wang CJ Arthroscopic reconstruction of the posterior cruciate ligament reconstruction with use of a quadruple hamstring tendon graft with 3- to 5-year follow-up Arthroscopy 2006 22 762 770 10.1016/j.arthro.2006.03.020 16843813

[B4] Chen B Gao S Double-bundle posterior cruciate ligament reconstruction using non-hardware suspension fixation technique and 8 strand of autogenously hamstring tendons Arthroscopy 2009 25 777 782 10.1016/j.arthro.2009.01.017 19560642

[B5] Wu CH Chen AC Yuan LJ Chang CH Chan YS Hsu KY Wang CJ Chen WJ Arthroscopic reconstruction of posterior cruciate ligament by using a quadriceps tendon autograft: a minimum 5-year follow-up Arthroscopy 2007 23 420 427 10.1016/j.arthro.2006.12.011 17418336

[B6] Murase K Kumano K Mannouji T Yokoe S Kaneko K Irie K Okubo F Radiographical measurement of anteroposterior instability of the knee joint 1987 Tokyo Knee Joint Meeting for Study, Japanese 179 186

[B7] Harner CD Höher J Evaluation and treatment of posterior cruciate ligament injuries Am J Sports Med 1998 26 471 482 9617416 10.1177/03635465980260032301

[B8] Kohen RB Sekiya JK Single-bundle versus double-bundle posterior cruciate ligament reconstruction Arthroscopy 2009 25 1470 1477 10.1016/j.arthro.2008.11.006 19962075

[B9] Berg EE Posterior cruciate tibial inlay ligament reconstruction Arthroscopy 1995 25 69 76 7727015 10.1016/0749-8063(95)90091-8

[B10] Wind WM Bergfeld JA Parker RD Evaluation and treatment of posterior cruciate ligament injuries, Revised Am J Sports Med 2004 32 1765 1775 10.1177/0363546504270481 15494347

[B11] Stannard JP Riley RS Sheils TM MaGwin G Volgas DA Anatomic reconstruction of the posterior cruciate ligament after multiligament knee injuries Am J Sports Med 2003 31 196 202 12642252 10.1177/03635465030310020701

[B12] Kim SJ Choi CH Kim HS Arthroscopic posterior cruciate ligament tibial inlay reconstruction Arthroscopy 2004 20 149 154 15243450 10.1016/j.arthro.2004.04.023

[B13] Kim SJ Kim TE Jo SB Kung YP Comparison of the clinical results of three posterior cruciate ligament reconstruction techniques J Bone Joint Surg Am 2009 91 2543 2549 10.2106/JBJS.H.01819 19884425

[B14] Sherlock MF Otto D Antegrade tibial tunnel technique for posterior cruciate ligament reconstruction Arthroscopy 2008 24 1301 1305 10.1016/j.arthro.2008.05.022 18971063

[B15] Wong T Wang CJ Weng LH Hsu SL Chou WY Chen JM Chan YS Functional outcome of arthroscopic posterior cruciate ligament reconstruction: Comparison of anteromedial and anterolateral trans-tibia approach Arch Orthop Trauma Surg 2009 129 315 321 10.1007/s00402-008-0787-3 19034466

